# Perspectives of sedentary behaviour, physical activity and health messaging among people with fibromyalgia: a qualitative study

**DOI:** 10.1093/rap/rkag081

**Published:** 2026-08-08

**Authors:** Rachel Bolton, Fliss Smith, Jennifer S Lewis, Jennifer Pearson

**Affiliations:** School of Health and Social Wellbeing, Glenside Campus, University of the West of England, Bristol, UK; School of Health and Social Wellbeing, Glenside Campus, University of the West of England, Bristol, UK; School of Health and Social Wellbeing, Glenside Campus, University of the West of England, Bristol, UK; The Bath National Pain Centre, Royal United Hospitals NHS Trust Bath, Bath, UK; School of Health and Social Wellbeing, Glenside Campus, University of the West of England, Bristol, UK; The Royal National Hospital for Rheumatic Diseases and Brownsword Therapies Centre, Royal United Hospital NHS Trust Bath, Bath, UK

**Keywords:** fibromyalgia, sedentary behaviour, physical activity, rest, movement, qualitative, self-management

## Abstract

**Objectives:**

To explore how individuals with fibromyalgia (FM) perceive physical activity (PA), sedentary behaviour (SB) and related health messaging.

**Methods:**

UK-based adults (18+) with FM were recruited using maximum variation sampling from charities, social media support groups and research contacts. A constructivist qualitative approach was adopted, recognizing that experiences and meanings are constructed through social context and interpretation. Semi-structured interviews were conducted virtually, informed by a behavioural model of Capability, Opportunity and Motivation (COM-B) and the Theoretical Domains Framework (TDF). A patient and public involvement (PPI) contributor informed the development of the interview guide. Data were analysed using a reflexive thematic analysis to explore how participants interpreted SB, PA and health messaging.

**Results:**

Fifteen participants (13 female, 2 male) aged 21–73 years (mean = 50.87, SD = 15.47) described complex, often conflicting experiences of PA and SB. Emotional responses like guilt, fear, and frustration influenced engagement with PA. Rest and activity were viewed as beneficial; however, balancing movement and rest was an ongoing challenge, influenced by fluctuating symptoms and external expectations. Participants highlighted a disconnect between clinical advice and lived experience, with terms like ‘sedentary’ often perceived as unrelatable or stigmatizing. Socioeconomic factors, including limited access to personalized support and social rules, impacted engagement and contributed to feelings of stigma.

**Conclusion:**

Participants reported emotional and practical challenges in balancing PA and SB, influenced by fluctuating symptoms, expectations and limited or unrelatable clinical advice. These findings highlight the need for personalized, inclusive, and context-sensitive movement advice that reflects lived experience to improve relevance and uptake.

Key messages

- The benefit of physical activity is recognized by people with fibromyalgia, but participation is shaped by complex personal factors and actual or anticipated symptom exacerbation.
- The need to rest is intertwined with perceptions of sedentary behaviour, often leading to guilt and unrest.
- Interactions with clinicians profoundly influence self-management and self-efficacy, and foster either feelings of support or distrust.

## Introduction

Fibromyalgia (FM) is a chronic condition characterized by complex symptoms, including widespread pain, fatigue, sleep disturbances and reduced physical function [1]. It affects approximately 5.4% of the UK population, with a higher prevalence in women [2]. The pathophysiology of FM is poorly understood; however, central sensitization is widely accepted as a key mechanism [3].

Physical activity (PA) and exercise interventions have robust evidence supporting their effectiveness in reducing FM symptoms [4, 5]. Several systematic literature reviews highlight the effectiveness of aerobic and strength training in symptom management and illness severity reduction [4, 5]. Despite this, individuals with FM often exhibit low adherence to moderate-to-high-intensity PA [6], where moderate intensity is defined as 50–70% of maximum heart rate (HRmax) and high intensity as ≥70% HRmax [7], and report high levels of sedentary behaviour (SB), characterized by activities involving ≤1.5 METs in a sitting, reclining or lying posture [8]. These individuals face unique barriers to activity engagement, including fluctuating symptoms, fear of exacerbation and uncertainty about how to exercise safely [9].

Independent of PA levels, habitual SB is associated with adverse health outcomes such as obesity, type 2 diabetes and heart disease [10, 11]. These risks may be heightened in FM, where SB is linked to increased pain, disability and disease impact [12–15]. Consequently, some research has explored reducing SB as a more acceptable and sustainable approach for this population, with lower intensity activity often preferred to avoid symptom exacerbation [16].

Qualitative research has highlighted the emotional, social and practical challenges that influence engagement with movement in FM. A recent qualitative evidence synthesis identified themes of fear, loss, stigma and the struggle to balance activity with symptom management, emphasizing the importance of personalized and context-sensitive support [9]. Other qualitative studies similarly suggest that PA is often deprioritized by people with FM due to symptom burden, with decisions influenced by fear, loss and anxiety [17]. However, little is known about how people with FM perceive their own activity patterns, the continuum between SB and PA, or the influence of health messaging and broader contextual factors. Therefore, this study aimed to explore how people with FM understand and navigate SB, PA and the messaging surrounding them. Understanding these perspectives is essential for developing interventions that are both effective and acceptable.

This study draws on the behavioural model of Capability, Opportunity and Motivation (COM-B) and the Theoretical Domains Framework (TDF) to explore the behavioural influences on PA and SB in people with FM [18, 19]. These frameworks provide a lens for understanding the capabilities, opportunities and motivations underpinning behaviour, incorporating the social and environmental domains that impact decision-making. By drawing on these models the research aims to highlight the contextual and experiential factors that influence engagement across SB and PA.

We aimed to explore: the knowledge and understanding of SB and PA from the perspective of people with FM; participants’ views on the gap between SB and PA; and the perspective and understanding of messaging around SB and PA in people with FM.

## Methods

### Methodological approach

This research adopted a constructivist qualitative approach, recognizing that experiences and meanings are constructed through social context, interpretation and interaction. This epistemological stance is well suited to exploring how individuals make sense of PA, SB and related health messaging, as it emphasizes the subjective and contextual nature of lived experience. The approach supports an interpretive analysis focused on understanding how participants described and interpreted their experiences, rather than seeking underlying causal mechanisms or objective truths. This constructivist stance aligns with reflexive thematic analysis, which emphasizes the co-construction of meaning between researcher and participant and recognizes the active, interpretive role of the researcher in theme development [20].

### Participants

A maximum variation sampling strategy was used to recruit UK-based participants from FM charities, social media support groups and existing research contacts. This ensured diversity across key characteristics including age, gender and length of diagnosis. Inclusion criteria were: adults aged 18 years or older, living in the UK, with either a self-reported or clinical diagnosis of FM, including those with co-morbidities. Exclusion criteria were: individuals without FM, those unable to communicate in English without support from carers and individuals requiring a translator for spoken or sign language, as translation services were not available for this study.

A target sample size of 14–16 participants was established to capture diversity in experiences relevant to the study aims. This target was informed by the concept of information power, whereby studies with a focused aim, specific sample, strong quality of dialogue and established theoretical frameworks require fewer participants to generate rich and meaningful data [21]. The sample size was therefore considered sufficient to support in-depth exploration of experiences while maintaining analytic depth. The lead researcher (R.B.) had no prior relationship with any participants before recruitment. Potential participants were contacted directly by R.B. after expressing interest through charity advertisements, social media posts or research networks. All communication prior to interview was limited to providing study information, answering questions and arranging interview times, ensuring that the researcher–participant relationship was neutral at the outset of data collection.

### Data collection

A semi-structured interview topic guide (Supplementary Data S1, available at *Rheumatology Advances in Practice* Online) informed by existing research [9, 16, 17] and drawing on the COM-B model [18] and the TDF [19] was developed to explore behavioural influences on PA and SB. These frameworks were used to ensure coverage of relevant behavioural domains during guide development but did not structure coding or theme development. The interview guide was used flexibly to support open exploration of participants’ experiences but was not iteratively adapted to refine or confirm theoretical propositions. This guide was refined in collaboration with a patient and public involvement (PPI) contributor, enhancing data collection and ensuring relevance to the FM population. The PPI contributor, who had lived experience of FM, provided feedback on the clarity, tone and relevance of the interview questions. This involved reviewing the draft topic guide and participating in two feedback discussions with the research team. Their input informed the wording, sequencing and sensitivity of questions to ensure they were accessible and meaningful for participants. PPI involvement focused on the development of the interview guide and did not extend to data analysis. All participants provided written informed consent prior to the interview and verbally confirmed their consent at the start of the interview. Interviews were conducted virtually by the lead researcher (R.B.) using Microsoft Teams and recorded. With participant permission, the interviews were conducted as video calls and were recorded through the platform; however, only the audio component was used for transcription and analysis. This approach facilitated UK-wide participation and accommodated accessibility needs while ensuring privacy and data security. Interview recordings were automatically transcribed using Microsoft Teams. Formal field notes were not taken during interviews; however, R.B. wrote brief reflective notes immediately afterwards to capture initial impressions, contextual details and emerging thoughts. These reflections were revisited during coding and supported reflexive engagement with the data. R.B. also discussed emerging ideas and analytic direction with her academic supervisor throughout the process. The transcripts were subsequently anonymized and reviewed to ensure accuracy and preserve the richness of the participants’ accounts, then uploaded to NVivo 14 for qualitative data management and analysis [22].

### Data analysis

Data were analysed using a constructivist, reflexive thematic analysis, recognizing that meanings are co-constructed through interaction and interpretation [20]. Transcripts were reviewed, any corrections were made, and then the transcripts were anonymized by the lead researcher. This supported familiarization with the data. Four transcripts, selected to reflect variation across the sample, were initially coded inductively without reference to existing frameworks. Early codes and developing analytic ideas were then discussed and refined in consultation with a senior clinical academic researcher (J.P.) to support interpretive rigour. The remaining transcripts were coded iteratively, with R.B. moving between data, codes and developing interpretations as analysis progressed [23]. Coding was conducted iteratively, with ongoing refinement of categories and concepts as analysis progressed. Analysis was ongoing throughout, and based on the data collected, no new themes were being identified in the final few interviews. NVivo 14 supported data management and coding throughout the process [22].

### Ethical considerations

This study was reviewed and approved as a low-risk project, with ethical oversight delegated to the module supervisors in accordance with the University of the West of England Faculty Research Ethics procedures. According to the Health Research Authority (HRA) decision-making tool, this study did not require NHS Research Ethics Committee approval because it involved non-NHS participants. The research was conducted in accordance with the principles outlined in the Declaration of Helsinki and adhered to good governance, data protection and General Data Protection Regulation (GDPR). All patients provided informed consent prior to taking part.

### Reflexivity

The lead researcher (R.B.) approached this study as a newly qualified physiotherapist and early-career qualitative researcher. At the time of data collection, R.B. had limited clinical experience of working with people with FM, which shaped the construction of data in several ways. This early-career positionality meant entering interviews with openness, curiosity and few entrenched assumptions, while drawing on emerging professional knowledge of movement, behaviour and health communication. R.B. was mindful of how this background influenced rapport, questioning style and interpretation, and engaged in ongoing reflexive practice throughout the study.

At the same time, the supervisory team included an experienced clinical academic researcher (J.P.), whose expertise in FM provided ongoing support and reflection on how R.B.’s emerging clinical identity and developing understanding of FM shaped data interpretation. These supervisory discussions offered opportunities to explore alternative interpretations, challenge assumptions and ensure transparency in analytic decision-making.

Reflexive notes were written immediately after each interview to capture initial impressions, emotional responses, assumptions and early analytic thoughts. These notes were revisited during coding to support awareness of how R.B.’s positionality and developing understanding influenced interpretive decisions. This reflexive process aligned with the constructivist stance adopted in the study, recognizing that knowledge was co-produced through interaction between researcher and participants, and that R.B.’ s positionality formed an integral part of the analytic process.

## Results

### Sample demographics and characteristics

A purposive sample of 20 individuals was invited to participate. Of these, two declined and three did not respond, resulting in a sample of 15 participants (13 female, 2 male), aged 21–73 years (mean = 50.87, SD = 15.47). Seven participants had been diagnosed for ≤2 years and eight for >2 years. See Table 1 for details.

**Table 1:** Participant characteristics.

Participant characteristics		Number	Percentage %
Gender	Female	13	87
	Male	2	13
Age	18–39 years	3	20
	40–59 years	7	47
	60+ years	5	33
	Mean (SD)	50.87 (15.47)	
	Range	21–73	
Length of diagnosis	≤ 2 years	7	47
	> 2 years	8	53

### Interview findings

Interviews lasted an average duration of 49.5 min. Analysis of the data generated four key themes: experiences of SB and PA; balancing the space between rest and movement; terminology, messaging and advice; and socioeconomic factors—each with associated subthemes. These themes reflect participants experiences and perspectives and are presented below with illustrative quotes. For a more comprehensive overview of the themes and illustrative quotes, please see Supplementary Table S1, available at *Rheumatology Advances in Practice* Online.

### Conceptual clarification

Consistent with the study’s constructivist approach, participants interpreted ‘sedentary behaviour’, ‘rest’, ‘physical activity’ and ‘activity’ in diverse and overlapping ways, shaped by their individual experiences and social contexts. This reflects the multiple subjective meanings these concepts held for people with FM, where the boundaries between these behaviours are fluid and often determined by personal interpretation, symptom fluctuation and external expectations [24]. Table 2 illustrates how these concepts were understood and applied by participants, highlighting the overlap and distinctions that emerged throughout the interviews.

**Table 2:** Illustration of overlap and distinctions between perceptions of SB, rest, PA and activity.

Task/action	Sedentary behaviour (SB)	Rest	Physical activity (PA)	Activity	Notes/participant perspective
Watching television	✓	**✓**		**✓**	Sedentary, often restful, but mentally active for some
Reading	**✓**	**✓**		**✓**	Sedentary, restful, but mentally active
Napping in waking h	**✓**	**✓**			Sedentary and restful
Sitting at a desk (working)	**✓**			**✓**	Sedentary, not restful but considered activity
Gardening			**✓**	**✓**	Physical activity and activity that is enjoyable and mentally active
Walking			**✓**	**✓**	Sometimes considered as activity but not physical activity
Crafting	**✓**	**✓**	**✓**	**✓**	Was referred to in relation to all categories
Housework			**✓**	**✓**	Physical activity or activity. Not seen as restful
Lying or reclined while awake	**✓**	**✓**			Seen as sedentary with negative connotations or restful

### Theme 1: experiences of SB and PA

The central organizing concept of this theme is the way participants made sense of movement and rest through an ongoing negotiation between bodily sensations, emotional responses and personal expectations. This theme explores internal beliefs, emotions and motivational factors that shaped engagement with PA and patterns of SB.

#### Subtheme 1.1: conflicting beliefs around benefits and risks

Despite recognizing the benefits of PA, most participants reported limited engagement. A few described regaining tolerance through consistent, graded activity and viewed PA as a form of management of symptoms. SB was seen as necessary and a beneficial part of managing symptoms, promoting rest, and supporting mental well-being, particularly when it involved purposeful or meaningful activities that were mentally stimulating such as crafting.

P006 ‘*I think doing nothing for your brain is sometimes is very good. But for your body it’s not*’.

However, both PA and SB were described as conditional and having potential benefits and risks, with no clear dichotomy between ‘good’ and ‘bad’ behaviours.

#### Subtheme 1.2: emotional responses to activity and rest

Participants described complex emotional responses to both activity and rest. Feelings of guilt, embarrassment and self-blame were common, particularly when rest was perceived as unproductive or when activity conflicted with social expectations or personal responsibilities.

P009: ‘*Half-way through a walk… I know I’m going to get punished for it… this is kind of your fault*’

These negative emotions often acted as internal barriers, influencing how participants navigated and justified their engagement with both behaviours.

#### Subtheme 1.3: motivation and self-belief

Motivation to engage in PA was impacted by a dynamic interplay of internal drivers and external conditions. While some participants drew on intrinsic motivators like independence and self-worth, others relied on external cues such as weight changes or seasonal shifts.

P015: ‘*It’s a let-down…I’m going to aim for that walk… I just can’t do it…then youlet down on yourself*’

Self-belief played a pivotal role, with motivation often disrupted when goals were not met, highlighting the emotional complexity of sustaining activity. This illustrates how motivation and self-efficacy are impacted by unmet goals.

### Theme 2: balancing the space between rest and movement

This theme captures how participants navigated the shifting boundary between rest and movement, responding to fluctuating symptoms, expectations and energy levels. This theme illustrates how individuals interpret and manage the relationship between SB and PA in everyday life, including how they understand and apply movement-related guidance. The continuum between rest and movement was discussed in relation to pacing and activities that participants described as active, but not PA.

#### Subtheme 2.1: approaches to pacing

Pacing was widely understood as a strategy to prevent the boom-and-bust cycle, but its application varied. Physically active participants used pacing alongside gradual increases in activity to build capacity, meaning they gradually improved their ability to tolerate and perform physical tasks over time. In contrast, less active individuals used pacing primarily to balance daily responsibilities with rest.

P006: ‘*Today I have ten spoons, tomorrow might only have three, and I have to use wisely*’.

Aiming to preserve energy and manage symptoms, rather than improve function, energy was seen as a limited and precious commodity—reflecting a more conservative, adaptive pacing approach.

#### Subtheme 2.2: keeping moving

Participants often viewed everyday tasks, such as housework, shopping and childcare, as legitimate forms of PA, though performed out of necessity rather than intention. Gardening stood out as a more enjoyable and acceptable form of exertion. While pain sometimes prompted gentle movement, fatigue led to rest. Some participants used creative strategies, like personifying their condition to foster acceptance and maintain activity.

P012: ‘*I said, come on fibro, we can do this… it’s a way of dealing with the pain level*’.

This demonstrates how less conventional strategies for PA engagement and imaginative coping mechanisms can help individuals engage with movement.

### Theme 3: terminology, messaging and advice

This theme highlights how participants evaluated and interpreted the language and advice surrounding PA and SB, shaping their sense of what felt meaningful, achievable and personally relevant. This theme illustrates how individuals interpret and respond to movement-related messaging, including the ways terminology and advice shape their understanding of PA and SB. Participants discussed the terminology used around PA and SB, the relatability of messaging and the nature of the advice they received.

#### Subtheme 3.1: terminology and communication

Participants reflected on the language used to describe PA and SB. While ‘physical activity’ was generally accepted, terms like ‘movement’ or ‘keeping active’ felt more relatable. ‘Sedentary behaviour’ was often perceived as stigmatizing, with alternatives like ‘resting’ or ‘inactivity’ preferred. The core concern was not terminology alone, but the lack of clear, tailored guidance on what these concepts should look like in practice.

P009: ‘*I do understand that exercise and movement is good… but I don’t know where the balance lies. There’s no education… should I be running? Should I be walking?*’

This illustrates how unclear messaging, and a lack of tailored guidance can negatively impact engagement, leaving individuals uncertain about how to participate meaningfully in PA.

#### Subtheme 3.2: lack of relatable or inclusive messaging

While some found online support groups helpful, many participants encountered challenges in finding messaging that felt relevant or hopeful. Overly negative narratives contributed to feelings of disconnection, while those who shared success stories to inspire others were often dismissed as unrelatable.

P007: ‘*“it’s irrelevant, you’re walking up mountains.” But, I started from not being able to do anything as well, I do know how it feels*’

This reflects the complexity of finding hope without alienation.

#### Subtheme 3.3: advice

Participants frequently reported a lack of clear, supportive guidance from healthcare professionals. Clinical materials, such as the FM leaflet provided through their local healthcare services, were often seen as outdated or unrelatable, failing to reflect participants’ age or lived experience. Advice was commonly vague, dismissive or overly focused on rest, with little emphasis on progression or potential for improvement. Some described feeling overwhelmed when seeking self-directed information, while others experienced medical gaslighting (having their symptoms dismissed or minimized by healthcare professionals) and disparities in care.

P001: ‘*It responds differently to everybody. Look it up on the internet. And that was the advice I was given*’.

Participant narratives revealed how vague and impersonal advice, often to self-directed research, can leave individuals feeling unsupported and overwhelmed.

### Theme 4: socioeconomic factors

This theme illustrates how socioeconomic circumstances shaped participants’ capacity to engage with movement, influencing both the opportunities available and the constraints they navigated. This theme highlights how structural and socioeconomic contexts influence individuals’ interpretations of, and ability to act on, movement-related guidance. Socioeconomic factors were apparent across all aspects, with employment and financial stability having both a positive and negative impact on the ability to engage with PA and reduce SB.

#### Subtheme 4.1: financial and employment barriers

Participants’ engagement with PA was influenced by financial pressures and employment demands. While work limited time and energy for PA and reinforced sedentary habits due to cultural norms in the workplace, it was also seen as more active than being unemployed and provided a sense of routine and purpose. Structured or paid activities, such as personal training, were often perceived as more beneficial than free activities such as walking, offering reassurance through professional guidance and decision-making.

P004: ‘*I had to cancel the gym because money is a bit tight at the minute… Now I’m trying to motivate myself to do the classes, but it’s like how do you do them at home?*’

An important implication of these findings is how socioeconomic status (SES) not only impacted access but also perceived legitimacy of different forms of PA.

#### Subtheme 4.2: FM in society

Participants described a lack of societal understanding of FM, contributing to stigma, social isolation and reduced confidence. Misconceptions from others and social norms often pressured some individuals to conceal symptoms reinforcing invisibility. Reports around aids were mixed whereby some tried to avoid using them due to them reinforcing disability stereotypes, whereas others valued them as a tool to enable participation.

P003: ‘*We can’t even go on the train together, we have to go separate compartments…cause there’s no there’s not space for two wheelchairs…so if you’re asking about physical activity and things like that, society’s not ready for us*’.

These factors influenced how participants navigated public spaces and social roles, influencing their engagement with PA and broader social inclusion.

## Discussion

This study aimed to explore how people with FM understood SB, PA and the messaging surrounding them. The findings address this aim by showing how participants made sense of PA and SB, the terminology around them and the advice and messaging they received. Participants’ experience with PA and SB revealed that a general understanding of the benefits and risks was evident; however, engagement was influenced by a range of emotional and behavioural responses. This is consistent with findings from a path analysis study that showed while individuals with FM may understand the benefits of PA, engagement is influenced by emotional and psychological factors [25]. For some, PA was associated with fear of symptom exacerbation, frustration and self-blame. These findings suggest that clinicians may need to acknowledge these emotional responses explicitly and provide reassurance and personalized guidance that supports confidence and gradual re-engagement with movement. Others, often those who had previous experience of PA, appeared to cope more effectively. They still experienced similar tensions but described greater flexibility and a sense of hope or belief in their ability to improve and manage symptoms over time. This aligns with findings from broader chronic illness research whereby past experience and behavioural patterns established before diagnosis are predictors of future engagement in PA [26]. Other factors, such as having goals for the future and higher self-efficacy and confidence in their ability to be active, helped participants engage more positively with PA and SB. Previous research has shown that being able to regulate emotions and having a positive self-perception can support ongoing participation in PA [27].

Rest, while seen as necessary, was often accompanied by guilt and perceived failure. These emotionally charged decisions, influenced by internalized stigma, sometimes led to maladaptive patterns such as avoidance [27]. This aligns with research describing psychological conflict and pain-related fear in FM, which reduce PA adherence, and with evidence that internalized stigma influences self-perception and behavioural choices [28].

Participants’ accounts reflected domains within the COM-B and TDF models, particularly reflective motivation, psychological capability and emotion. However, they are primarily present-focused and do not explicitly consider past behaviours or experiences as predictors of future engagement [29, 30]. This gap overlooks the role of pre-illness behaviours and habits, which, if integrated, could offer valuable insight for designing more effective behaviour change strategies in FM.

Participants described the ongoing challenge of balancing rest and movement, often using pacing strategies to manage symptoms, which was frequently interpreted as energy conservation rather than a structured, goal-oriented strategy to improve function. This reflects a broader misunderstanding noted in the literature, where pacing is applied adaptively (in response to symptoms) rather than operantly (to build function), limiting functional gains [31]. Analogies like the ‘spoon theory’, though relatable, are not evidence-based and may reinforce symptom-contingent behaviours rather than support progression. Clarifying the purpose of pacing and supporting individuals to develop strategies that promote functional progression, rather than solely symptom avoidance, may help improve engagement and self-efficacy.

Applied to the COM-B and TDF, these interpretations reflect limited psychological capability (e.g. understanding of pacing principles), reflective motivation, beliefs about consequences and emotion and behavioural regulation, as the goal is often pain avoidance rather than functional improvement. Although research supports the benefits of pacing, these findings suggest symptom-contingent strategies are dominant, highlighting the need for more widespread messaging [32].

Some participants externalized FM through personification, which seemed to align with behavioural regulation in the TDF, as it allowed negotiation with symptoms rather than feeling out of control. While not widely studied in FM, this aligns with broader psychological models that support pain acceptance as coping strategies in chronic illness, helping individuals to regulate emotion and maintain value-driven behaviour [33].

Participants valued non-stigmatizing language, preferring terms like ‘movement’ over ‘physical activity’ and ‘inactive’ over ‘sedentary behaviour’; however, the core concern was a lack of clear, practical guidance. Generic information, including clinical materials such as leaflets, often failed to reflect the heterogeneity of FM, particularly around age, lifestyle or lived experience [34]. This disconnect contributed to disengagement, highlighting gaps in psychological capability and environmental context within the COM-B model and TDF. Research supports the need for tailored communication in complex, variable conditions like FM to improve relevance, trust and behavioural outcomes [34, 35]. Improving the clarity, relatability and personal relevance of movement-related messaging may help bridge the gap between clinical advice and lived experience, supporting more meaningful engagement.

Messaging was often vague, outdated or overly focused on rest, with little emphasis on progression or potential for improvement, undermining reflective motivation and beliefs about capabilities. Participants also noted a lack of hopeful, relatable narratives. Conversely, success stories intended to inspire were often dismissed or minimized as unrelatable, reflecting the tension between hope and alienation in chronic illness communication. This was compounded by experiences of medical gaslighting where symptoms were dismissed or minimized, leading to mistrust, disengagement, and reduced self-efficacy [36, 37].

Socioeconomic deprivation influenced participants’ engagement with PA and SB, with those with financial stability better able to prioritize health. Financial constraints and employment demands, limited access to structured PA and reinforced sedentary routines. This is consistent with evidence that lower SES increases symptom severity and increases functional impairment, which both impact on PA adherence and SB [38]. Participants viewed paid activities as more legitimate, reflecting how SES influences beliefs about what constitutes as valid PA. Financial and social opportunity domains of the COM-B were constrained, while motivation factors were undermined by stigma and societal misunderstandings of FM contributing to social pain which impacted on participants’ ability to engage in meaningful activity [30]. The TDF domains of social influences, environmental context and beliefs about capabilities linked with societal norms around productivity and disability, which further marginalized participants and resulted in social isolation, with aids sometimes seen as reinforcing negative stereotypes [39]. These findings underscore the need for inclusive, low-cost PA interventions that address structural barriers and challenge societal narratives around disability.

### Strengths and limitations

This study benefited from a maximum variation sample and the involvement of a PPI contributor in developing the interview guide, ensuring relevance to the FM population. Importantly, participants discussed PA and SB based on their own interpretations, offering authentic insights into lived perceptions rather than researcher-imposed definitions. A strength of the analysis process was the review of the coding framework in collaboration with a senior researcher, which helped ensure rigour. However, several limitations should be noted. All participants were social media users, potentially excluding those without digital access or literacy. Although purposive sampling was used to achieve variation across age, gender and length of diagnosis, ethnicity was not included as a sampling characteristic and demographic data on race were not collected. As a result, the sample lacked ethnic diversity, which may limit the transferability of the findings to under-represented groups. Future research should consider incorporating ethnicity within purposive sampling strategies to explore how cultural experiences may shape engagement with PA, SB and health messaging in FM. PA and SB levels were self-reported, and no objective activity data were recorded. SES was estimated using postcodes to generate Index of Multiple Deprivation (IMD) scores. This was limited by the fact that the IMD applies only to England and some participants did not provide full postcodes, resulting in incomplete SES data. This restricts how far the findings can account for wider socioeconomic influences.

## Conclusion

This study highlights how emotional responses, fluctuating symptoms and the nature of clinical advice impact on how people with FM navigate PA and SB. These findings address the research question by highlighting the need for personalized, context-sensitive guidance around rest and movement in FM care. Guidance that accounts for not only physical and social factors but also for the complex emotional and psychological dimensions of living with an often-misunderstood condition. Drawing on behavioural frameworks like the COM-B and TDF helps to identify the complex interplay of emotional, psychological and contextual factors, such as internalized stigma, fluctuating self-efficacy and the influence of past experiences, shaping engagement with PA and SB in FM.

Future research should prioritize co-designed approaches, involving a diverse group of people with lived experience to inform interventions that are both meaningful and grounded in the real-world challenges and successes of those living with FM. Additionally, scaling up pacing education—potentially via accessible platforms such as social media—may help shift perceptions while reinforcing empowering, relatable narratives around self-management.

## Supplementary Material

rkag081_Supplementary_Data

## Data Availability

The qualitative data generated and analysed during this study are not publicly available due to confidentiality and ethical restrictions associated with patient participants. Anonymized quotes supporting the findings are included within the article. Further information may be available from the corresponding author upon reasonable request and subject to appropriate ethical review.

## Notes

*Disclosure statement:* The authors have declared no conflict of interest.
