# Supplementary material for: Perspectives of sedentary behaviour, physical activity and health messaging among people with fibromyalgia: a qualitative study
Source: Rheumatol Adv Pract. 2026 Aug 8;10(3):rkag081. doi: 10.1093/rap/rkag081 (PMC13456962; doi:10.1093/rap/rkag081)
Supplement: Supplementary file 1 [file rkag081_supplementary_data.docx]

**Supplementary Table S1: Illustrative participant quotes**

| **Subtheme** | **Participant Quote** | **Participant ID** |
| --- | --- | --- |
| 1.1: Conflicting beliefs around benefits and risks | *“So, I've got different forms of sedentary, so one is the pain is so tiring. The lack of sleep or the fatigue that's hitting so that that's. That follows with reducing major body movements … or there’s the bits where I need to rest my body but I've got other movements I can do, so I will do.*  *I'll keep need to keep my brain active”.* | P002 |
|  | “*I think it's self-regulating because I'm always wanting to be more active. Rather than wanting to be inactive. But I will want to be inactive when I'm hurting*” | P002 |
|  | *“I mean the main activity is probably taking the kids to school in the morning…And it's not that far. I know it needs to be increased. I do and I think it would probably help my symptoms. I've got the literature. It's just mind over matter, isn't it at some point and time?”* | P005 |
|  | *“There is certainly benefits from having those times of rest”.* | P001 |
|  | “*I just know that physical activity will help and it does - 10/10 times it does - might not be as brilliant whilst I'm doing it, but it does help*” | P012 |
| 1.2: Emotional responses to activity and rest | *“It's so embarrassing, you know, to be out with your mates and then going. God, I'm in so much pain. So you cut yourself off at home. You cancel events, you don't go out”.* | P004 |
|  | “*It still feels lazy... you’re not doing what you should be doing*” | P005 |
|  | *“The guilt around rest and I think there's a lot. I mean, maybe even as a child, we're productive, aren't we? What's next? What are we doing next? What have I achieved today?”* | P007 |
| 1.3: Motivation and self-belief | “*I'll try and do a little bit at home,* *but I’m not very disciplined at doing that at home... I’ve got no willpower”* | P001 |
|  | *“I think it's just the end of the day and I just need to switch off. That's not the time to be then going out doing a physical activity. I wish it was. I wish I had the energy and the motivation at that point”* | P005 |
|  | *“last year I thought, well, the weight's piling on. Maybe we should try the gym. So I've been experimenting ever since”* | P011 |
|  | *“I think it's the challenge of doing, of being able to do it with fibro. I think to me it's like I'm not going to be beaten. You know, we're going to do this together and I'm really not going to let it define me. I'm going to, you go with it. All right? I've. I've taken a hit afterwards, but.*  *Yeah. It's just that sense of self achievement really.”* | P012 |
| 2.1: Approaches to pacing | *“the spoon theory, so everybody at the end of the day counts how many spoons we've got left because everybody starts off with a different amount …Every now and again … I've just run out of spoons. A couple of times I've just sat on the stairs, even getting up the stairs and I'm like, I'm done. My spoons have gone”.* | P005 |
|  | *“I did it (a gym course) for the whole 12 weeks and it was only an hour in the gym, say every other day. What they didn't see either side of that picture was me on the settee having to pace myself. So I have to pace myself an awful lot”.* | P010 |
|  | *“When I'm sort of resting and pacing at home and not doing too much, but I find if I sit on the sofa for ages, I'll get achy. So I'm one who, I'll get up and move, and it does help me”.* | P011 |
|  | *“Some people think it's really bad and that they have to be, go, go, go all the time. But that is not healthy for someone with this disability. You know, like the boom and bust cycle was way too easy to get stuck into….So how do I pace? I listen to my body. I'm pretty good at knowing like when I need to, like, stop doing something”.* | P013 |
| 2.2:Keeping moving | *“I just try and do as much as I can, just going out in the wheelchairs, is a bit of an exercise, you know, with the pavements and things like that nowadays. So that's that's exercise”* | P003 |
|  | *“I've got an allotment and I absolutely love my allotment and that is hard going. People think you're just putting some plants in, you know, last weekend we were building a greenhouse … Then I think you get a lot of like, mental pleasure from it. You know, planting things, watching it grow. But you do get lots of physical activity, like doing your beds and getting weeds out. …So for me, am I getting my steps in, no.*  *It'll ping up on the app saying, you know. Keep going. Keep trying to get those steps in for the day, but actually I'm doing lots of other physical activity and lots of things that's good for your mental health. Being outside, getting your hands in the dirt, like that's really good”.* | P009 |
|  | *“All the non-gym physical activity, definitely gardening. Yeah, lots of it. So, I’ve just been... laying bark chippings last week and that was hard work”* | P011 |
| 3.1: Terminology and communication | *“I think maybe instead of saying, don't be still or sedentary, get them do something. Do you have any hobbies? You know, all right. You're in pain. You can't move your arms today. What about tomorrow? Do you know? Do you have a raised plant bed? To find something that somebody's interested in and to take away”.* | P006 |
|  | *“I suppose I have negative connotations of it (SB). I see it as being critical of somebody and pretty much calling them lazy I suppose.*  *Yeah, I guess to rephrase it to like. I mean, I don't mind movement break. So I guess rather than say sedentary is negative, to sell the positive”.* | P007 |
|  | *“as a whole wide enveloping sort of term, yes. But I take it to more of a specific level, but keeping active, you know, even if you're sort of sitting in the chair and move, you know, just getting up and moving is physical activity… I think there needs to be more sort of guidance really on, you know what physical, what that term suggests really”.* | P012 |
| 3.2: Lack of relatable or inclusive messaging | “So I've got access to their materials and talk to fellow sufferers that have fibromyalgia and we share best tips. And I honestly in all the things I join in, I think peer-to-peer is incredibly powerful. You we learn more peer-to-peer than we do from our medics in a lot of cases”. | P002 |
|  | *“I joined the fibromyalgia (support group) website and I know it's a site for people ranting, but I go on there and I just think, Oh my gosh… where's the positivity in this group? So I actually stopped reading it. I just thought, I don't want to go down that route. That's not for me”.* | P006 |
| 3.3: Advice | *“That's ageist and dismissive and patronising, like, like seeing someone for my breathlessness and they asked for a description or when are you getting more breath? Well, I used to be able to do 10 squats without getting puffed out. Now I'm really puffing and gasping by the end of that, and they laughed and they said, Oh well, I think if I tried 10 squats, I'd be breathless as well.*  *And afterwards I thought, yes but have you actually had a personal trainer for 10 years? You know, what is your starting point? …And the other remark, you're quite good for your age. Don't look at my what's written down on the paper in terms of my date of birth, look at me. The person. Listen to what I'm saying. So that made me very angry”.* | P014 |
|  | *“I didn’t really feel like I related to it (patient leaflet) at all... all the case studies were about women in their 50s, 60s, 70s. At the time I was 18”* | P008 |
|  | *“They want you to slow down”* | P003 |
|  | *“It’s not like you’ll ever be running around the gym”* | P011 |
|  | *“When they thought it was arthritis... I was offered all these treatments. With FM, it was like, you’re on your way”* | P015 |
| 4.1: Financial and employment barriers | *“I think poverty brings in fibromyalgia as well…if you lose your job today.*  *And you have nothing else in place, and you're on your own. You'll end up with £79 a week to live on, you know, to pay for everything. You'll spend more than that in the supermarket in half an hour”.* | P003 |
|  | *“There’s a gym nearby that I’m trying to get the money for”* | P013 |
| 4.2: FM in Society | *“there's that added thing from an exercise perspective of you know, how do you. How do you do what you need to do when society is really very, very difficult to deal with? If you don't follow a structured pattern or a trend or whatever”* | P004 |
|  | *“I think that's sort of probably the hardest thing in getting other people to understand how it affects you. You know, my mother seems to think that it's like arthritis or that that it's affected by the weather, you know, how’s your needs today? Well, my knees are OK, you know, how’s all your aches and pains? Well, aching and painful. Oh, yeah, well, it's the weather, isn't it? No, no, no, no, it's not the weather. It just hurts, you know. You know, a lot of people don't understand”.* | P001 |

**Supplementary Data S1.**

**Perspectives and Understanding of Sedentary Behaviour and Physical Activity among people with Fibromyalgia: A qualitative study**

**Semi Structured Interview Topic Guide**

Introduction – We’re going to discuss your thoughts around balancing activity, movement, rest and time spent sitting, in general and in relation to Fibromyalgia. It will include gaining your insight into some common terms used to describe these activity levels and your thoughts on these.

| **Question** | **Rationale** | **Prompt** | **COM-B** |
| --- | --- | --- | --- |
| What does physical activity (PA) mean to you? | To gain understanding around participants knowledge of the concept of PA. (Objective 1) | Can you give examples of ways in which you would say people are physically active?  Would you say there are any activities that you do that are physically active? | Capability – Psychological |
| A term often used to describe low levels of activity is sedentary behaviour (SB). Can you tell me what this term means to you? | To gain understanding around participants knowledge of the concept of SB. (Objective 1) | Can you give examples of ways in which you would say people engage in sedentary behaviour?  On a typical day, what activities do you engage in that would be considered low levels of activity or rest? | Capability – Psychological |
| What are your thoughts on the balance between physical activity and rest or sitting time and their impact on health? | To gain understanding around impact on health in general of PA and SB. (Objective 1) | What is your knowledge around the risks associated with not getting enough physical activity? If people don’t do enough physical activity, does that put them at risk of certain health conditions or diseases? If so, what are they?  Are there any risks or benefits associated with sedentary behaviour. Do high levels of SB or sitting put people at risk of certain health conditions? If so, what are they? | Capability – Psychological  Capability – Psychological |
| What sort of things make it difficult to be physically active?  What sort of things help you to be physically active? | To determine how these terms are viewed in relation to FMS. (Objective 1) | Does your FMS impact on your ability to be active. If so, in what ways?  Are there any other aspects of life such as family, work, home or in society that affect your activity levels? | Capability – Physical capability  Opportunity – Physical and social |
| Is it difficult to limit sedentary behaviours such as sitting for long periods?  What sort of things help to reduce sedentary time? | To determine how these terms are viewed in relation to FMS. (Objective 1) | Does FMS affect your ability to reduce your sitting time?  Are there any other aspects of life such as family, work, home or in society that affect your sedentary behaviour? | Capability – Physical capability  Opportunity – Physical and social |
| Is balancing sitting time and physical activity important to you? If so, why? | To determine the relationship and balance between these two concepts. (Objective 2) | What are some ways that you could break up sitting time?  Are there things that people do in sitting that could be done in standing?  Do you rest following physical activity?  What types of activities do you feel would fall between the two activity levels of sedentary behaviour and physical activity. | Motivation – Reflective  Opportunity – Social and Physical  Motivation – Reflective and automatic  Motivation – Reflective |
| Have you come across the terms physical activity and sedentary behaviour before when interacting with health care professionals? | To determine the messaging around SB and PA and what that means to participants (Objective 3). | In what instance were these terms used? What do you feel was the intent of the messaging?  Were they helpful?  What other terms do you feel would have been more appropriate or motivating to you when that person was conveying the message? | Opportunity – Social  Motivation – reflective |
